# Transmission of MDR and XDR Tuberculosis in Shanghai, China

**DOI:** 10.1371/journal.pone.0004370

**Published:** 2009-02-03

**Authors:** Ming Zhao, Xia Li, Peng Xu, Xin Shen, Xiaohong Gui, Lili Wang, Kathryn DeRiemer, Jian Mei, Qian Gao

**Affiliations:** 1 Department of TB Control, Shanghai Municipal Center for Disease Control and Prevention, Shanghai, China; 2 Key Laboratory of Medical Molecular Virology, Institutes of Biomedical Sciences and Institute of Medical Microbiology, Fudan University, Shanghai, People's Republic of China; 3 School of Medicine, University of California Davis, Davis, California, United States of America; University of Cape Town, United Kingdom

## Abstract

**Background:**

Multidrug-resistant (MDR) and extensively drug-resistant (XDR) tuberculosis (TB) are global health problems. We sought to determine the characteristics, prevalence, and relative frequency of transmission of MDR and XDR TB in Shanghai, one of the largest cities in Asia.

**Methods:**

TB is diagnosed in district TB hospitals in Shanghai, China. Drug susceptibility testing for first-line drugs was performed for all culture positive TB cases, and tests for second-line drugs were performed for MDR cases. VNTR-7 and VNTR-16 were used to genotype the strains, and prior treatment history and treatment outcomes were determined for each patient.

**Results:**

There were 4,379 culture positive TB cases diagnosed with drug susceptibility test results available during March 2004 through November 2007. 247 (5.6%) were infected with a MDR strain of *M. tuberculosis* and 11 (6.3%) of the 175 MDR patients whose isolate was tested for susceptibility to second-line drugs, were XDR. More than half of the patients with MDR and XDR were newly diagnosed and had no prior history of TB treatment. Nearly 57% of the patients with MDR were successfully treated.

**Discussion:**

Transmission of MDR and XDR strains is a serious problem in Shanghai. While a history of prior anti-TB treatment indicates which individuals may have acquired MDR or XDR TB, it does not accurately predict which TB patients have disease caused by transmission of MDR and XDR strains. Therefore, universal drug susceptibility testing is recommended for new and retreatment TB cases.

## Introduction

Since 1994, nearly 90 countries and regions worldwide have reported one or more cases of multidrug-resistant (MDR) tuberculosis (TB) [1]. MDR TB is defined as TB caused by a strain of *M. tuberculosis* that is resistant to at least isoniazid and rifampin, two of the most important first-line drugs used to treat the disease [2]. China had approximately 140,000 MDR TB cases in 2004, or one third of the estimated global burden of MDR TB [1]. In some provinces of China, the prevalence of MDR TB among new cases and previously treated cases was above 10% and 30%, respectively [3]. However, the number and percentage of MDR TB cases in Shanghai, one of the largest cities in Asia, has not previously been reported.

In 2006, multiple cases of extensively drug-resistant (XDR) TB were reported in South Africa, raising concerns that XDR strains of *M. tuberculosis* are prevalent in other populations. Many of the cases reported from South Africa occurred among HIV-infected persons and there was a high case fatality rate [4]. By November 2007, 41 countries had reported XDR TB cases [5]. The mortality rate of XDR TB patients varies in different countries and depends on the study population and their HIV status [4], [6]–[8]. More than fifty percent of HIV-uninfected XDR TB patients have been successfully treated in South Korea [6], but the duration of treatment is prolonged and the financial burden is high [7]. XDR TB has not previously been formally reported in mainland China, but given the high prevalence of TB nationwide, the large number of MDR TB cases in the country, and the long-time use of second line anti-TB drugs and fluroquinolones, the existence of XDR TB in China seems inevitable.

We performed a retrospective study to determine the number and percentage of TB patients in Shanghai with MDR and XDR TB, and to determine whether there is transmission of MDR and XDR strains of *M. tuberculosis* in Shanghai.

## Materials and Methods

### Study population

We performed a retrospective cohort study using the existing data and specimens at the Shanghai Municipal Center for Disease Control and Prevention (Shanghai CDC), from TB patients who were diagnosed in Shanghai during March 2004 through November 2007. Since 1995 in Shanghai, all suspected pulmonary TB cases detected in general hospitals or community health centers were referred to a specialized TB hospital or TB clinic for further diagnostic tests, including sputum smear examinations, culture and chest radiography. There were 31 designated district TB hospitals in Shanghai; all of the pretreatment positive cultures from patients in each hospital were sent to the Tuberculosis Reference Laboratory (TRL) at Shanghai CDC for drug susceptibility testing and species identification. Shanghai CDC also collected data on the social and demographic characteristics, treatment history, clinical characteristics, drug-susceptibility test results, and clinical outcomes of each patient. All of the investigation protocols in this study were approved by ethics committee of Fudan University. Since this was a retrospective study and all patients' information was routinely collected by Shanghai CDC for analysis and reports to the government, consent was not obtained from the patients during 2004 through 2006. We started a research project in 2007, and informed consent has been obtained from all patients since then for the information to be used in scientific studies.

### Drug susceptibility testing (DST)

TRL at Shanghai CDC participated in the World Health Organization/International Union against Tuberculosis and Lung Disease Global Project on Anti-Tuberculosis Drug Resistance Surveillance [2]. Species identification of mycobacteria was performed by conventional biochemical and PCR tests [9]. The first-line drug susceptibility testing was routinely performed by the absolute concentration or proportion methods on isolates of *M. tuberculosis* [10].

In the present study, we restricted our analysis to those patients whose first-line drug susceptibility tests were performed by the proportion method. The following drug concentrations were used: isoniazid (0.2 µg/ml), rifampin (40.0 µg/ml), streptomycin (4.0 µg/ml) and ethambutol (2.0 µg/ml). For any isolates that were MDR, we also used the WHO Guidelines for drug susceptibility testing for second-line anti-tuberculosis drugs for DOTS plus [11]. We chose five second-line drugs widely used for MDR TB treatment in Shanghai and performed drug susceptibility tests using the following concentrations: ofloxacin (2.0 µg/ml), kanamycin (30.0 µg/ml), capreomycin (40.0 µg/ml), amikacin (40.0 µg/ml) and 4-aminosalicylic acid (1.0 µg/ml). To ensure the consistency and reliability of results, all second-line drug susceptibility testing was performed by a senior technician who had completed and passed the WHO's quality control evaluation. All drugs were obtained from Sigma Life Science Company (USA).

### Definitions

MDR TB was defined as tuberculosis disease caused by a strain of *M. tuberculosis* that was resistant to at least isoniazid and rifampin. XDR was defined as TB with resistance to at least isoniazid, rifampin, a fluoroquinolone (e.g. mofloxacin, ofloxacin, levofloxacin, sparfloxacin, gatifloxacin, ciprofloxacin) and one of three injectable second-line drugs (capreomycin, kanamycin, and amikacin) [12]. Pre-XDR was defined as disease caused by a strain resistant to isoniazid and rifampin and either a fluoroquinolone or a second-line injectable drug, but not both [13]. In the present study, we use the terms simple MDR, which refers to isolates that are resistant to just isoniazid and rifampin but not pre-XDR TB and XDR TB.

New cases were defined as TB patients who denied having had any prior anti-TB treatment or who received anti-TB treatment for <30 days. Previously treated cases were TB patients who reported having been treated for tuberculosis for at least 30 days or who had documented evidence of prior treatment in the case report form or surveillance database. Acquired drug resistance was defined as the isolation of drug-resistant *M. tuberculosis* from a patient who has been treated for tuberculosis for one month or longer [14]. Primary drug resistance is the isolation of a drug-resistant strain from a patient without a history of previous treatment [14].

Migrants were defined as individuals from other areas of China who moved to Shanghai. Residents were defined as persons with a registered permanent residence in Shanghai.

### Genotyping method

We used the VNTR-7 and VNTR-16 methods to genotype the 189 clinical isolates of *M. tuberculosis*, following the protocol described by Zhang [15]. First, VNTR-7 was performed in all isolates and the isolates with identical VNTR-7 genotyping pattern were further differentiated by VNTR-16. We also used the deletion-targeted Multiplex PCR (DTM-PCR) method to identify the Beijing genotype strains [16]. Primers were synthesized by Invitrogen Bio Co. (China) and polymerase chain reaction (PCR) mixtures were prepared using the 2×Taq MasterMix (Tiangen Co., China). PCR products were separated using an agarose gel and were analyzed by Quantity 1 gel imaging system (Bio-Rad Co., USA).

### Clinical treatment

There is presently no standard treatment strategy, such as DOTS-Plus, to guide the therapy of MDR-TB patients in Shanghai. Individualized therapies were given to MDR TB patients based on the patient's physical and financial situation, the strains' drug-susceptibility patterns and the clinicians' experience. The following drugs were used, in different combinations: two injectable second-line drugs, including capreomycin and amikacin; fluoroquinolones, including ofloxacin, levofloxacin, gatifloxacin, moxifloxacin and ciprofloxacin; a modified form of isoniazid, called prothionamide; two modified forms of rifampicin, called rifapentine and rifabutin; and 4-aminosalicylic acid.

### Statistical analysis

We used the chi-square test of proportions to identify significant differences between two or more groups of patients. A *p* value<0.05 was considered statistically significant. Odd ratios (ORs) and 95% confidence intervals (CI) were calculated to measure the association between patient characteristics and the outcome of interest. All analyses were performed using Stata statistical software (version 8.0SE, Stata Corporation, College Station, Texas, USA).

## Results

### MDR TB

**Figure 1:**
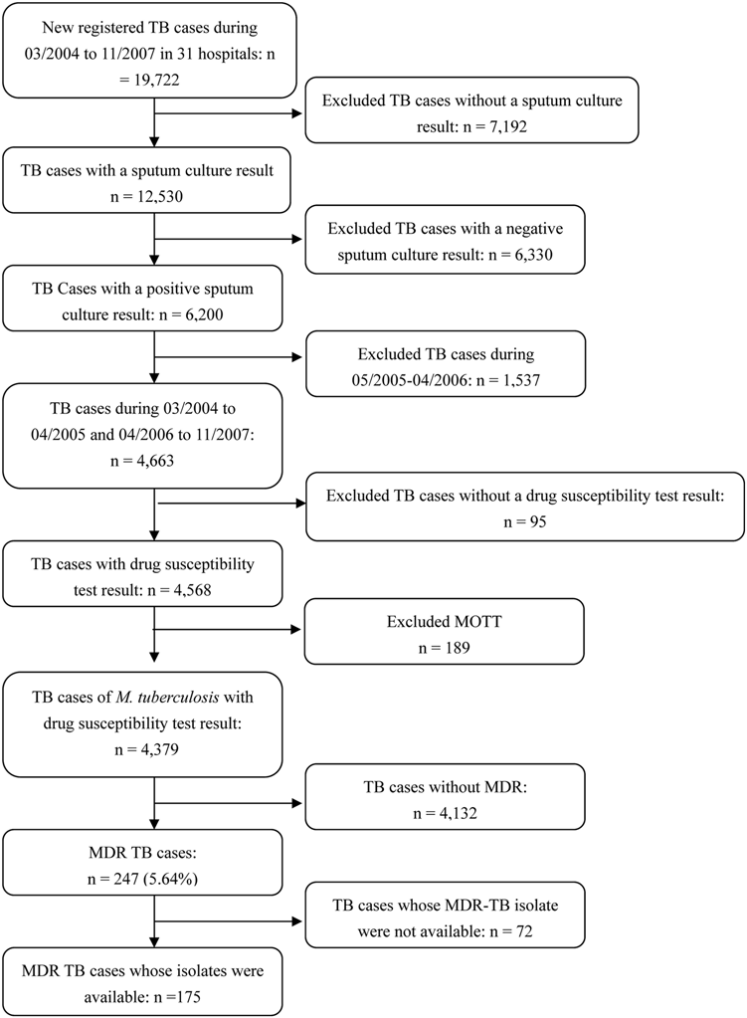
Study population of cases of drug resistant tuberculosis in Shanghai, March, 2004–November, 2007. TB = tuberculosis. MDR = multidrug-resistant. MOTT = Mycobacteria other than tuberculosis.

From March 2004 through November 2007, there were 19,722 newly registered pulmonary tuberculosis patients in 31 designated district tuberculosis hospitals in Shanghai. Of these, 6,200 (31.4%) patients were culture positive for *M. tuberculosis*. We excluded 1,537 culture-positive TB patients who were diagnosed during May 2005 through February 2006 because the drug susceptibility testing of their isolates was performed using the absolute concentration method. We compared the characteristics such as sex, age and treatment history of the patients who were included and excluded from the study population and found no significant differences (data not shown). Of the remaining 4,663 TB patients, we further excluded 95 (2.0%) patients whose isolate lacked a drug susceptibility test result and 189 (4.1%) patients who were infected with Mycobacterium other than *M. tuberculosis* (MOTT). The remaining 4,379 patients with culture-confirmed *M. tuberculosis* and their drug susceptibility test results were used for analysis. Among them, 247 (5.6%, 247/4,379) TB patients had disease caused by a MDR strain of *M. tuberculosis* (figure 1).

### XDR TB

To determine the number and percentage of the MDR TB patients that were infected with an XDR strain of *M. tuberculosis*, we performed drug susceptibility testing for five second-line drugs on the isolates of the MDR TB patients in the study population. However, the isolates from 72 of the 247 MDR TB patients were missing or could not be recovered from the storage; isolates from 175 (70.9%) MDR TB patients were tested for susceptibility to a second-line drug. We compared the clinical characteristics of the TB patients whose isolate was tested for susceptibility to second-line drugs and the TB patients whose isolates were missing or could not be recovered, and there were no statistically significant differences between the two groups (data not shown). Among the 175 MDR patients, 109 patients (62.3%) were infected with a strain of *M. tuberculosis* that was simple MDR, 55 patients (31.4%) were infected with a strain that was pre-XDR, and 11 (6.3%) patients were infected with an XDR strain of *M. tuberculosis*.

### Characteristics of MDR, pre-XDR and XDR TB patients

We compared the patient characteristics associated with MDR, pre-XDR and XDR TB, such as age, sex, treatment history and status (resident versus migrant) (table 1). Most MDR patients were 30 to 59 years old, and XDR TB was more likely to occur in persons 45 to 59 years than among person who were younger or older (p = 0.0067). Sixty percent of the patients with MDR and 54.5% of the patients with XDR were new cases.

**Table 1:** Characteristics of tuberculosis patients by drug resistance group, Shanghai, March 2004–November 2007.

Characteristic	All MDR	Simple MDR	Pre-XDR	XDR	χ^2^	P
	n = 175 (%)	n = 109 (%)	n = 55 (%)	n = 11 (%)		
Age (years)					16.182	0.040
15–29	35 (20.0)	24 (22.0)	11 (20.0)	0 (0.0)		
30–44	53 (30.3)	37 (33.9)	13 (23.6)	3 (27.3)		
45–59	57 (32.6)	26 (23.9)	23 (41.8)	8 (72.7)		
60–74	20 (11.4)	14 (12.8)	6 (10.9)	0 (0)		
≥75	10 (5.7)	8 (7.4)	2 (3.6)	0 (0)		
Sex					2.772	0.250
Male	132 (75.4)	84 (77.1)	38 (69.1)	10 (90.9)		
Female	43 (24.6)	25 (22.9)	17 (30.9)	1 (9.1)		
Treatment history					1.314	0.519
New	105 (60.0)	69 (63.3)	30 (54.5)	6 (54.5)		
Retreatment	70 (40.0)	40 (36.7)	25 (45.5)	5 (45.5)		
Status					0.480	0.787
Resident	112 (64.0)	70 (64.2)	34 (61.8)	8 (72.7)		
Migrant	63 (36.0)	39 (35.8)	21 (38.2)	3 (27.3)		

MDR = resistance to at least isoniazid and rifampin.

Simple MDR = resistance to only isoniazid and rifampin.

Pre-XDR = Pre-extensively drug resistant; the strain is resistant to isoniazid, rifampin, and a fluoroquinolone or three of the second-line drugs (capreomycin, amikacin, kanamycin).

XDR = extensively drug resistant; the strain is resistant to isoniazid, rifampin and a fluoroquinolone and any of three of the second-line drugs (capreomycin, amikacin, kanamycin).

### Treatment outcomes of MDR patients

Fifty-six percent of the patients with MDR TB and 9.1% of the patients with XDR TB were successfully treated. The cure rate in simple MDR TB, pre-XDR TB and XDR TB patients decreased and the mortality increased as the drug resistance increased (table 2). The cure rate of MDR TB in new cases was higher than the cure rate of MDR TB among previously treated cases (table 3).

**Table 2:** Treatment outcome of tuberculosis patients with MDR TB, pre-XDR TB, and XDR TB.

	Simple MDR	Pre-XDR	XDR	Total
	n = 109 (%)	n = 55 (%)	n = 11 (%)	n = 175 (%)
Cured, bacteriological confirmed	62 (56.9)	29 (52.7)	1 (9.1)	92 (52.6)
Completed treatment regimen	3 (2.8)	2 (3.6)	0 (0.0)	6 (3.4)
Died during TB treatment	6 (5.5)	5 (9.1)	1 (9.1)	12 (6.9)
Still on treatment	30 (27.5)	14 (25.5)	8 (72.7)	51 (29.1)
Lost to follow up	1 (0.9)	1 (1.8)	1 (9.1)	3 (1.7)
Moved/transferred	7 (6.4)	4 (7.3)	0 (0)	11 (6.3)

MDR = resistance to at least isoniazid and rifampin.

Simple MDR = resistance to only isoniazid and rifampin.

Pre-XDR = Pre-extensively drug resistant; the strain is resistant to isoniazid, rifampin, and a fluoroquinolone or any one of three second-line drugs (capreomycin, amikacin, kanamycin).

XDR = extensively drug resistant; the strain is resistant to isoniazid, rifampin and a fluoroquinolone and any one of three second-line drugs (capreomycin, amikacin, kanamycin).

**Table 3:** Treatment outcomes among tuberculosis (TB) patients with simple MDR TB, pre-XDR TB and XDR TB, stratified by new cases versus previously treated cases.

	Treatment success	No treatment success	Odds ratio (OR)	95% CI	P
	N = 96 (54.9%)	N = 79 (45.1%)			
XDR					
New	0 (0.0)	6 (3.4)			
Retreatment	1 (0.6)	4 (2.3)	-	-	0.2506
Pre-XDR					
New	23 (13.1)	9 (5.1)	1.00		
Retreatment	8 (4.6)	15 (8.6)	4.79	(1.32, 17.85)	0.0062
Simple MDR					
New	47 (26.9)	19 (10.9)	1.00		
Retreatment	17 (9.7)	26 (14.9)	3.78	(1.56, 9.24)	0.0010
Total					
New	70 (40.0)	34 (19.4)	1.00		
Retreatment	26 (14.9)	45 (25.7)	3.56	(1.80, 7.06)	0.0001

OR = odds ratio.

CI = confidence interval.

MDR = resistance to at least isoniazid and rifampin.

Simple MDR = resistance to only isoniazid and rifampin.

Pre-XDR = Pre-extensively drug resistant; the strain is resistant to isoniazid, rifampin, and a fluoroquinolone or any one of three second-line drugs (capreomycin, amikacin, kanamycin).

XDR = extensively drug resistant; the strain is resistant to isoniazid, rifampin and a fluoroquinolone and any one of three second-line drugs (capreomycin, amikacin, kanamycin).

### Genotypes of *M. tuberculosis*

We genotyped one isolate from each of 175 MDR TB patients. 87.3% (165/189) of the MDR isolates and 90.9% (10/11) of the XDR isolates were Beijing genotype strains. Patients infected with *M. tuberculosis* strains that had the same VNTR pattern were assumed to be part of a chain of transmission [15], [17]. Four clusters were identified, each with 2 different patients. To determine whether the cluster of two patients represented a chain of transmission of *M. tuberculosis*, we sought epidemiological links between them. Patients in two clusters lived in different districts, but patients in the other two clusters lived in the same district and had been diagnosed and treated in the same hospital and no additional epidemiological links between them were detected. None of the isolates with XDR were in a genotype cluster.

## Discussion

The present study showed that 5.6% of the tuberculosis patients in Shanghai were infected with a strain of *M. tuberculosis* that was MDR. Furthermore, 6.3% of the MDR TB patients were XDR. Nearly 55% (6/11) of the XDR TB patients were new cases, suggesting there is transmission of highly drug-resistant strains of *M. tuberculosis* in Shanghai.

Since second-line drugs have been used in Shanghai for several decades and there is no standard treatment strategy for patients with MDR and pre-XDR strains, there was concern that the prevalence of XDR TB in Shanghai would be higher. Based on our study using specimens from the 31 designated district tuberculosis hospitals, we report that XDR TB occurs in Shanghai, albeit currently with a relatively low prevalence during the study period.

After XDR TB became a public concern during 2006, many countries retested their stored isolates and XDR strains were reported in 41 countries [5]. The reported percentage of XDR TB patients among the MDR TB patients varies between countries, from 3% in the United States to 19% in Latvia [18], and the average rate is about 10% [19]. The percentage of MDR and XDR TB patients that are detected depends on the study design, the sampling frame and the study population. For example, hospital based studies will likely include a greater proportion of seriously ill TB patients, including patients who are undergoing retreatment. In contrast, community based studies will likely provide a lower estimate of the percentage of total TB patients who are infected with an MDR or XDR strain of *M. tuberculosis*.

More than half of the infections with MDR and XDR strains of *M. tuberculosis* in our study occurred among new TB cases, a finding which suggests that the transmission of MDR and XDR strains of *M. tuberculosis* is a serious problem in Shanghai. The prevalence of primary drug resistance may actually be higher than our estimate. Transmission of drug-resistant *M. tuberculosis* can cause primary drug resistance among individuals with no prior history of TB, as well as among individuals with a history of prior TB treatment. Previously, we showed that 84% of retreated TB patients with drug-resistant disease actually had primary drug resistance, not acquired drug resistance [20]. Andrews, et al. also reported that most of the MDR/XDR cases in South Africa were primary drug resistance [21]. In addition, a recent meta-analysis showed that primary drug resistance was associated with poor treatment outcomes if treatment regimens were not based on drug susceptibility test results [22]. In communities with a high rate of primary drug resistance, approaches which assign treatment regimens based solely on the patient's history of prior treatment may amplify drug resistance [22]. MDR and XDR patients in China are often poor and cannot afford the second-line anti-TB drugs, making it difficult to achieve treatment success. MDR and XDR patients without adequate treatment regimens will continue to be sources of infection, causing more MDR and XDR infections and TB patients in their communities. Therefore, new strategies, including rapid drug susceptibility testing techniques, are urgently needed.

To identify chains of transmission of *M. tuberculosis* is arduous work, requiring a prospective population-based cohort study design, high case detection rates and additional epidemiological information. In the present study, four clusters of patients with identical genotype patterns were identified among 175 MDR TB patients, but epidemiological links between them were difficult to establish. Another limitation of the present study is that isolates of some TB patients were not available for drug susceptibility testing and genotyping, or their drug susceptibility test was performed with the absolute concentration method. However, this is a retrospective study and we were able to investigate only some of the MDR TB patients. Therefore, the recent transmission of *M. tuberculosis* based on genotyping results is likely underestimated.

Overall, the treatment outcomes of MDR TB patients have been less favorable than the treatment outcomes of TB patients whose disease is caused by a pan-susceptible strain, a mono-resistant, or a poly-resistant strain of *M. tuberculosis* [23], [24]. Some MDR TB patients can be successfully treated, even as outpatients [25]. The XDR TB epidemic in Tugela Ferry, South Africa, which had a high prevalence of co-infection with HIV and a high case fatality ratio, led some to label XDR TB an “untreatable” disease. However, a study from South Korea reported that more than fifty percent of HIV-uninfected XDR TB patients were successfully treated [6], as were 60.4% of XDR TB patients in Peru and 41.2% of XDR TB patients completed therapy in California [13], [26].

Our study showed that the cure rate is higher among new TB cases than among previously treated TB cases, whether they were MDR or pre-XDR patients. A previous study reported that TB patients with primary drug resistant tuberculosis had better treatment outcomes than TB patients with acquired drug resistant TB [27], although this is not always the case. The concept of pre-XDR TB is important for public health purposes, to allow the community to assess how many XDR TB cases are likely to emerge and to make concerted efforts to treat the individuals whose disease is caused by a pre-XDR strain of *M. tuberculosis*. Theoretically, it is difficult to gain resistance to two kinds of drugs simultaneously. In our study, 78% (43/55) of the pre-XDR isolates were resistant to a fluoroquinolone and were, therefore, one mutation away from becoming XDR. Fluoroquinolones have been used extensively in Shanghai during the past twenty years to treat drug-resistant TB patients and retreatment patients.

In summary, 5.6% of the TB cases in Shanghai were infected with a MDR strain of *M. tuberculosis*, and further drug susceptibility testing showed that 6.3% of the patients whose isolate was MDR were actually XDR. More than half of the patients with MDR and XDR were new cases, suggesting that the transmission of drug-resistant strains in Shanghai is a serious problem. Currently, the rapid diagnosis and treatment of persons with TB, particularly any form of drug-resistant TB, are high priority public health interventions. Universal drug susceptibility testing is recommended for new and retreatment TB cases.
